# Structure and Diversity of Endophytic Bacteria in Maize Seeds and Germinating Roots

**DOI:** 10.3390/microorganisms12071348

**Published:** 2024-07-01

**Authors:** Yang Gao, Yujun Zhang, Puchang Wang, Lili Zhao

**Affiliations:** 1 Guizhou Academy of Agriculture Sciences, Guiyang 550025, China; gyphoebe945@126.com (Y.G.); zhangyj_92@163.com (Y.Z.); 2 School of Karst Science, Guizhou Normal University, Guiyang 550025, China; 3 College of Animal Science, Guizhou University, Guiyang 550025, China; 4 School of Life Sciences, Guizhou Normal University, Guiyang 550025, China; 5 State Key Laboratory of Microbial Technology, Shandong University, Qingdao 266237, China

**Keywords:** alpha diversity, co-occurrence network analysis, germinating roots, maize seeds

## Abstract

Seed endophytes in maize, which facilitate the transmission of microorganisms from one plant generation to the next, may play a crucial role in plant protection and growth promotion. This study aimed to investigate the effects of various maize varieties on the communities of endophytic bacteria in seeds and germinating roots. This study utilized Illumina high-throughput sequencing technology to examine the structural and diversity differences of endophytic bacterial communities within seed maize (BY1507), silage maize (QQ446), and wild maize (Teosinte) in both seeds and germinating roots. The results showed that 416 bacterial genera were detected, with *Pantoea*, *Lachnospiraceae*, *Pararhizobium*, *Enterobacteriaceae*, *Stenotrophomonas,* and *Pseudonocardia* being the most prevalent (relative abundance > 10%) at the genus level. No significant difference was observed in diversity indices (Chao1, ACE, Shannon, and Simpson) of seed endophytes among BY1507, QQ446, and Teosinte. The Shannon and Simpson indices for the germinating root endophyte from the wild variety (Teosinte) were significantly higher than the domesticated varieties (BY1507 and QQ446). PCoA revealed a notable overlap in the endophytic bacterial communities from the seeds of BY1507, QQ446, and Teosinte. Yet, clustering patterns were found. Co-occurrence network analysis showed that BY1507, QQ446, and Teosinte share a notable proportion of shared endophytic bacteria (>30%) between the seeds and germinating roots. This investigation elucidates the characteristics of endophytic microbial communities of seeds and germinating roots with seed maize, silage maize, and wild maize, offering data for future research on the physiological ecological adaptation of these endophytic microbial communities.

## 1. Introduction

Seeds play a fundamental role in plant reproduction, acting both as vehicles for genetic material and as adaptive mechanisms for species survival under environmental stresses. They are crucial for the continuation of plant species, significantly affecting reproductive success, progeny health, and agricultural productivity [1,2]. Extensive research has demonstrated that seeds are rich reservoirs of endophytic organisms [3,4]. Throughout the reproductive cycle, seeds enable the transfer of beneficial micro-organisms from parent plants to the offspring, influencing plant growth, development, productivity, and quality [5,6,7,8]. The seed microbiome, often referred to as the secondary genome in plant evolution, forms symbiotic relationships with host plants. These relationships evolve over time, contributing to vital physiological processes, such as energy metabolism, immune responses, and pathogen defense [9,10]. This symbiosis allows for the adaptive modulation of endophytes, helping plants navigate environmental changes and meet energy demands under stress conditions [11]. Seeds are known to serve as essential channels for microbial inheritance from maternal plants to offspring, showing significant heritability and environmental adaptability [12,13,14].

Bacteria are the most significant group of seed endophytes, playing essential roles in nutrient cycling, phytohormone production, and protection against biotic and abiotic stresses. These functions enhance plant establishment and resilience [2,7]. Recently, research has highlighted the critical role of seed endophytic bacteria in the survival and propagation of host plants [9,12]. The biotechnological applications of beneficial seed endophytic bacteria in agriculture and biological control, as well as the diversity and biological functions of these bacterial communities, have emerged as pivotal research areas [2,13]. Therefore, exploring the diversity and composition of seed endophytic bacteria and understanding the dynamics of these communities are crucial for uncovering mechanisms of species adaptation to their environments and for harnessing beneficial bacterial resources.

Maize (*Zea mays* L.), with its high photosynthetic efficiency due to the C4 metabolic pathway, is celebrated for its significant grain and biomass production [14]. As a major food and fodder crop worldwide, maize is cultivated across approximately 170 countries, covering an area of 194 million hectares and producing about 1.1476 trillion kilograms [15]. In China, maize accounts for one-third of the total major crop harvest area, boasting an annual output of more than 200 billion kilograms [15]. The study of interactions between maize seeds and their endophytic bacterial communities is crucial for improving yields and managing pests and diseases [16,17,18,19,20,21,22,23,24,25]. Advances in molecular biology and detection techniques have enhanced the sensitivity of endophyte identification in maize seeds, making the diversity of maize-seed endophytic bacteria a key research focus [26,27,28]. The diverse endophytic bacteria in maize contribute to a range of biological functions that enhance nitrogen fixation and promote maize growth [29]. Additionally, these bacteria possess biological control capabilities, effectively inhibiting pathogens and improving maize’s tolerance to stress [16,29,30]. Investigating this diversity is critical for understanding the bacterial community composition and variation in maize, paving the way for the identification and application of functional strains to improve maize productivity and health.

Modern maize, classified within the genus *Zea*, originated from the domestication of the wild annual tropical grass Teosinte in Southern Mexico approximately 9000 years ago [31]. Studies suggest that wild maize exhibits a higher resilience to environmental stressors compared to its modern counterparts, likely due to the presence of a more specialized endophytic community [31]. This leads to the hypothesis that the diversity of endophytes in maize seeds is affected by the traits of different varieties and that the transmission of endophytes from seeds to roots is both specific and viable. In light of this, our study selected three types of maize for investigation: seed maize (BY1507), silage maize (QQ446), and wild maize (Teosinte). Employing Illumina MiSeq DNA sequencing technology, we aimed to examine the diversity of endophytic microorganisms within the seeds and germinating roots of these maize varieties to test our hypothesis. The goal of this research is to lay a theoretical foundation for the identification of potential beneficial endophytes in maize seeds. Uncovering such endophytes is of strategic importance for boosting maize yields and enhancing its ecological and agricultural functions.

## 2. Materials and Methods

### 2.1. Materials

The plant seeds utilized in this study comprised seed maize (Beiyu 1507, BY1507), silage maize (Qianqing 446, QQ446), and wild maize (Teosinte). BY1507 and QQ446 were sourced from the Institute of Upland Food Crops, Guizhou Academy of Agricultural Sciences. Teosinte was sourced from the Maize Research Institute of Sichuan Agricultural University. All the seeds were collected in 2021. The experimental procedures were carried out in the Grassland Research Laboratory at the School of Life Sciences, Guizhou Normal University. To ensure experimental integrity, the seeds were stored in sterile bags, with each plant type represented by three replicates.

### 2.2. Pretreatment in Seeds and Roots

For seed pretreatment, the maize seeds from different sources were surface-sterilized [32] in three replicates, with 100 seeds per repeat. Initially, they were soaked in 70% alcohol for 5 min and then in a 2% sodium hypochlorite solution for 3 min, followed by 5–7 rinses with sterile water. One µL of the final rinse water from the seeds was spread on beef-extract peptone agar to check for sterilization effectiveness. After one week, if no colonies formed on the agar, it indicated complete surface sterilization of the seeds. These sterilized seeds were then used for DNA extraction. The more detailed information on seed surface sterilization method is described by Liu et al. [33].

For root pretreatment, the maize seeds from different sources were germinated in Petri dishes within a constant temperature light incubator set to a light intensity of 3000 lx, a 12 h photoperiod, and a temperature of 25 °C. After 14 days, the roots were sampled using the same method as above.

For sample collection, 5 g of seeds and 5 g of roots from each replicate were picked and rinsed with running water to remove surface dirt. The seeds (roots) were disinfected with a 2% sodium hypochlorite solution for 5 min, then washed multiple times with sterile water. The cleaned plants were placed in sterile EP tubes, approximately 5 g per replicate, with three replicates for each sample. The samples were stored in a −80 °C freezer, sealed, and sent to a company for the extraction of endophytic bacterial DNA from the seeds (roots) and Illumina MiSeq (Hiseq 2500 platform) sequencing.

### 2.3. Extraction of Endophytic Bacterial DNA from Seeds, Illumina MiSeq Sequencing, and Bioinformatics Analysis

The seed coat was cut open using sterile scissors under a sterile hood, and the internal tissue was transferred into a sterile mortar. After grinding thoroughly with liquid nitrogen, the material was transferred to the Power Beads tube of the DNeasy Power Soil Kit (QIAGEN, Inc., Venlo, The Netherlands) for microbial genomic DNA extraction. The DNA concentration and purity were measured using a Nano Drop ND-1000 (Thermo Fisher Scientific, Waltham, MA, USA), and the integrity of the DNA was analyzed using 0.8% agarose gel electrophoresis. The DNA samples were stored at −20 °C before PCR amplification. Primers 515F and 907R **[34]** were used to amplify the V4–V5 region of the bacterial 16S rRNA gene. Sample-specific 7 bp barcodes were incorporated into the primers for multiplex sequencing. The PCR reaction was carried out in a 25 μL volume, including 5 μL Q5 reaction buffer (5×), 5 μL Q5 high-fidelity GC buffer (5×), 0.25 μL Q5 high-fidelity DNA polymerase (5 U/μL), 2 μL dNTPs (2.5 mM), 1 μL of each forward and reverse primer (10 μM), 2 μL DNA template, and 8.75 μL ddH_2_O. The reaction conditions were 98 °C for 2 min; 25 cycles of 98 °C for 15 s, 55 °C for 30 s, 72 °C for 30 s; and a final extension at 72 °C for 5 min. PCR products were purified using Agencourt AM Pure Beads (Beckman Coulter, Indianapolis, IN, USA) and stored at −20 °C for sequencing. The PCR products were quantified using the Quant-IT PicoGreen dsDNA Assay Kit on a Microplate reader (Bio Tek, FLx800, Shoreline, WA, USA). The samples were pooled according to the required amount of data for each sample. Based on the quantification results, the samples were mixed in proportions corresponding to the sequencing requirements for each sample. Paired-end 2 × 300 bp sequencing was performed on the MiSeq platform using the MiSeq Reagent Kit v3 (Shanghai Parsonal Biotechnology Co., Ltd., Shanghai, China) [35,36]. The root tissue was transferred into a sterile mortar and then was sampled using the same method as above.

### 2.4. Data Processing

In this study, the abundance of specific taxa within each sample was inferred from the number of reads (or sequences), using this metric as an indicator of taxonomic abundance [36]. To mitigate the impact of potential sequencing artifacts, operational taxonomic units (OTUs) exhibiting a relative abundance of less than 1% across the entire dataset were excluded from further analysis. The methodology encompassed several critical steps, including the stitching and filtering of reads and clustering or denoising processes, followed by species annotation and abundance analysis to determine the species composition within the samples. The analysis of composition and richness distribution at the phylum and genus levels for each sample was conducted using Excel 97-2003 software [36]. To estimate bacterial diversity within the samples, known as alpha diversity, we utilized measures of richness (the number of observed OTUs) and Shannon’s diversity index. Differences in diversity between seeds and roots or among the varieties BY1507, QQ446, and Teosinte were evaluated using an analysis of variance (ANOVA) with SPSS 20.0 software [37]. Principal coordinate analysis (PCoA) was employed to visualize the patterns in the microbial community structure, or beta diversity, in seeds or roots among BY1507, QQ446, and Teosinte [38]. This analysis was based on the Bray–Curtis dissimilarities calculated from the log-transformed OTU abundances. Furthermore, the network co-occurrence analysis in this research was performed using a two-mode matrix, with the results visualized using COOC (Co-Occurrence 9.9) and VOSviewer (version 1.6.11) software [39]. This approach provided insights into the complex interactions and relationships within the microbial communities studied.

## 3. Results

The current study conducted a comprehensive examination of the bacterial composition within seed maize (BY1507), silage maize (QQ446), and wild maize (Teosinte) across both seeds and germinating roots. The dataset encompassed 18 samples, with the total number of sequences ranging from 136,827 to 153,553. On average, each sample yielded 148,610 effective sequences.

### 3.1. Composition of Endophytic Bacterial Communities in Seeds and Germinating Roots

**Figure 1:**
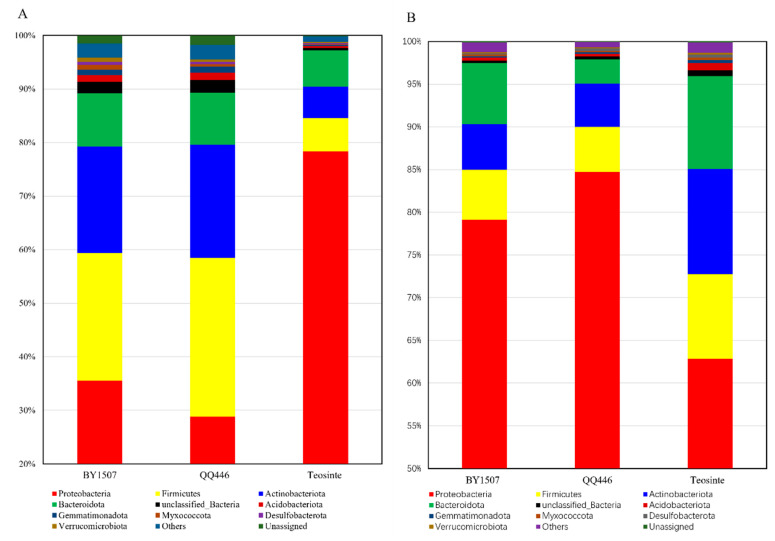
The structure of bacterial communities associated with seeds (**A**) and germinating roots (**B**) at phyla level among seed maize (BY1507), silage maize (QQ446), and wild maize (Teosinte).

The study uncovered a total of 18 bacterial phyla within the seeds and germinating roots of BY1507, QQ446, and Teosinte, showcasing the rich diversity of endophytic bacterial communities across these maize varieties (Figure 1). Predominant bacterial phyla, with a relative abundance exceeding 5%, included Proteobacteria, Firmicutes, Actinobacteriota, and Bacteroidota. Notably, the distribution of these phyla significantly differed among the maize varieties and between the seeds and roots. Seeds of BY1507 and QQ446 exhibited a higher abundance of Proteobacteria compared to Teosinte, suggesting a variety-specific association. In contrast, Firmicutes and Actinobacteriota were less abundant in the domesticated varieties (BY1507 and QQ446) than in Teosinte, indicating that domestication and selective breeding have likely impacted the bacterial composition. In germinating root samples, a contrasting trend was observed. The abundance of Proteobacteria was significantly higher in germinating roots compared to seeds for BY1507 and QQ446, while Firmicutes, Actinobacteriota, and Bacteroidota showed increased prevalence in the seeds. Teosinte seeds had a higher Proteobacteria abundance than roots, with a decreased presence of Firmicutes, Actinobacteriota, and Bacteroidota in germinating roots.

**Figure 2:**
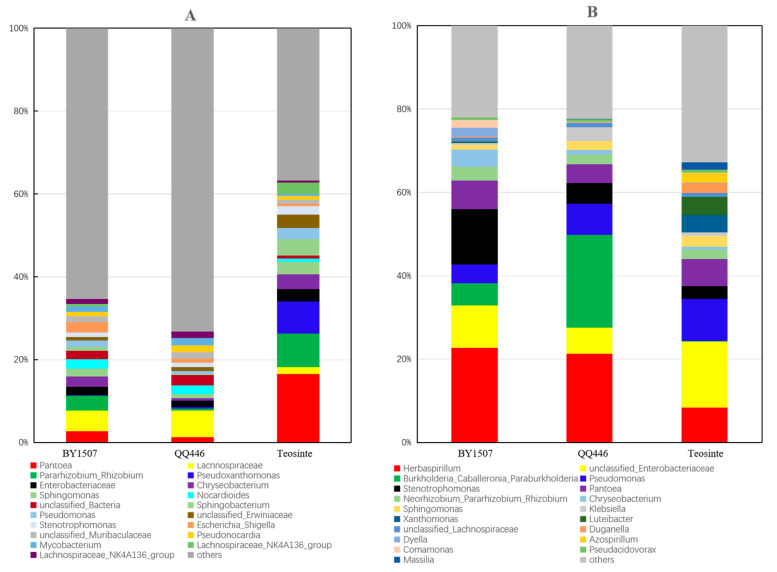
The structure of bacterial communities associated with seeds (**A**) and germinating roots (**B**) at genus level among seed maize (BY1507), silage maize (QQ446), and wild maize (Teosinte).

A genus-level analysis revealed a total of 416 genera in seeds, with 31 genera showing a relative abundance greater than 1% across all samples (Figure 2). Figure 2A highlights the diversity within the seed microbiome, presenting the most prevalent 20 bacterial genera. Among these, *Pantoea*, *Lachnospiraceae*, *Pararhizobium*, *Enterobacteriaceae*, *Stenotrophomonas*, and *Pseudonocardia* were consistently common across BY1507, QQ446, and Teosinte seeds. Dominant genera varied across maize varieties, with BY1507 characterized by *Pantoea*, *Lachnospiraceae*, and *Pararhizobium/Rhizobium*; QQ446 by *Lachnospiraceae*, *Nocardioides*, and unclassified_Bacteria; and Teosinte by *Pantoea*, *Pararhizobium*, and *Pseudoxanthomonas*. This demonstrates obvious differences in endophytic bacterial communities at the genus level among the varieties.

In germinating roots, the analysis identified the same 416 genera, with 31 genera having a relative abundance greater than 1% across samples, as depicted in Figure 2B. Seven genera were commonly found across BY1507, QQ446, and Teosinte roots: *Herbaspirillum*, unclassified *Enterobacteriaceae*, *Pseudomonas*, *Stenotrophomonas*, *Pantoea*, *Neorhizobium*, and *Sphingomonas*. The dominant genera in the root samples varied, underscoring significant genus-level differences in the endophytic bacterial communities of germinating roots among BY1507, QQ446, and Teosinte. This highlights the influence of plant variety on microbial diversity and community structure, emphasizing the complexity of endophytic bacterial interactions within maize.

### 3.2. Endophytic Bacterial Community Diversity in Seeds and Germinating Roots

#### 3.2.1. Alpha Diversity

**Figure 3:**
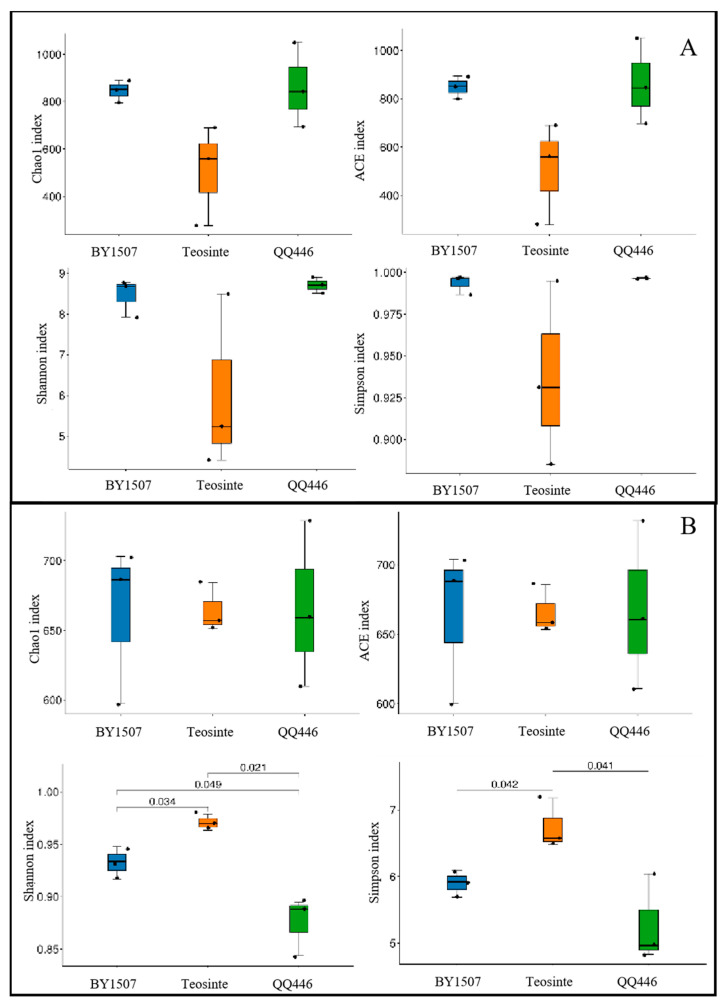
Differences in alpha diversity of bacterial communities associated with seeds (**A**) and germinating roots (**B**) among seed maize (BY1507), silage maize (QQ446), and wild maize (Teosinte).

This study’s alpha diversity analysis of endophytic bacterial communities within the seeds and germinating roots of BY1507, QQ446, and Teosinte utilized indices, such as Chao1, ACE, Shannon, and Simpson, to evaluate richness and evenness (Figure 3). The results indicated that there was no significant difference in the diversity indices of seeds among BY1507, QQ446, and Teosinte (Figure 3A). In contrast, Teosinte’s germinating roots exhibited a notably higher alpha diversity (Shannon and Simpson) compared to BY1507 and QQ446 (Figure 3B).

**Figure 4:**
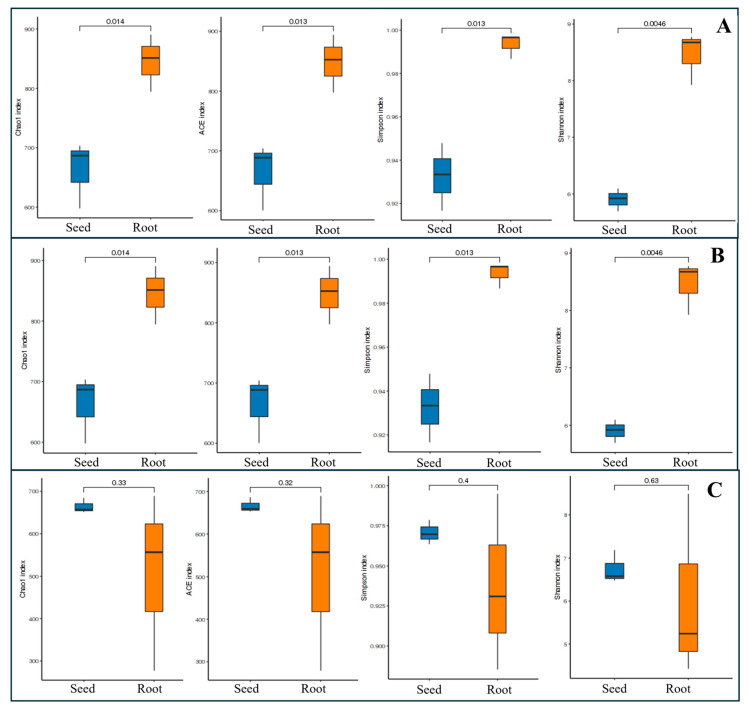
Differences in alpha diversity of bacterial communities associated with seeds and germinating roots among seed maize (BY1507) (**A**), silage maize (QQ446) (**B**), and wild maize (Teosinte) (**C**).

Further, the analysis demonstrated that the alpha diversity indices (Chao1, ACE, Shannon, and Simpson) for endophytic bacteria in BY1507 and QQ446 seeds were significantly lower than in their germinating roots (Figure 4). For Teosinte, no significant difference was observed in the diversity indices (Chao1, ACE, Shannon, and Simpson) between seeds and germinating roots.

#### 3.2.2. PCoA Analysis

**Figure 5:**
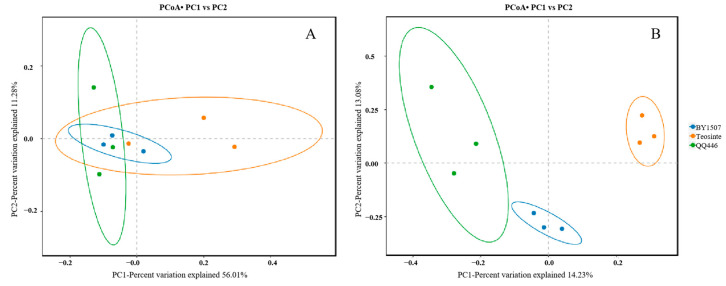
The beta diversity of bacterial communities associated with seeds (**A**) and germinating roots (**B**) among seed maize (BY1507), silage maize (QQ446), and wild maize (Teosinte).

The PCoA outcomes reveal a notable overlap in the endophytic bacterial communities from the seeds of BY1507, QQ446, and Teosinte (Figure 5). This overlap suggests a high degree of similarity in the microbial community structures within the seeds across these maize varieties. Conversely, the endophytic bacterial communities from the germinating roots of BY1507, QQ446, and Teosinte exhibit distinct clustering patterns in the PCoA analysis. This separation indicates significant differences in the composition of endophytic bacterial communities among the roots of these varieties.

### 3.3. Co-Occurrence Network of Endophytic Bacteria in Seeds and Germinating Roots

The co-occurrence network analysis sheds light on the intricate relationships between plants and their associated endophytic bacteria, providing insights into how these entities interact within the ecosystem. In this analysis, the nodes represent various endophytic bacteria and plant taxa, with the node size indicating the occurrence frequency of each plant and bacterial species. The thickness of the lines, or edges, signifies the strength of association between these entities.

**Figure 6:**
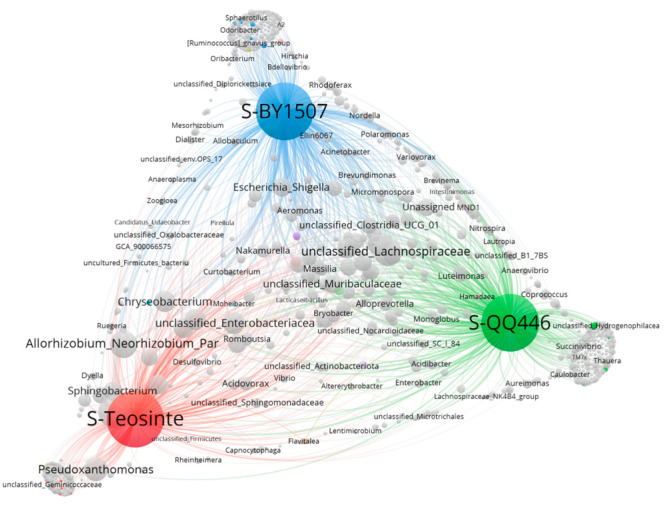
Co-occurrence network analysis of endophytic bacteria with seeds among seed maize (BY1507), silage maize (QQ446), and wild maize (Teosinte).

For the seeds of BY1507, QQ446, and Teosinte, the co-occurrence network (Figure 6) exhibited a complex structure comprising 809 genera or nodes connected by 2818 edges, organized into three main modules distinguished by different colors. This network revealed that 79 genera of endophytic bacteria, constituting 26.95% of all endophytes, were shared across the seeds of all three plant types. A total link strength (TLS) analysis highlighted Pantoea as having the highest connectivity within the seed endophytic bacterial community, followed by notable associations with unclassified_Lachnospiraceae, Allorhizobium_Neorhizobium_Pararhizobium_Rhizobium, Pseudoxanthomonas, and unclassified_Enterobacteriaceae. These findings underscore the pivotal roles of these genera, particularly Pantoea, in the microbial ecosystems of maize seeds.

The network analysis also identified numerous endophytic bacteria common across pairwise sharing (e.g., BY1507 and QQ446, BY1507 and Teosinte, and Teosinte and Teosinte), including *Plesiomonas*, *Roseburia*, *Succinivibrio*, *Rhodoferax*, and *Variovorax*. This commonality points to shared microbial associations among different maize varieties, suggesting a core set of endophytic bacteria that may play essential roles in seed biology across different plant species.

Moreover, the analysis revealed that each of the maize types—BY1507, QQ446, and Teosinte—harbors unique endophytic bacterial communities, further emphasizing the diversity of microbial life associated with maize and the potential for these unique endophytes to contribute to the distinct physiological and ecological traits of each plant type. This complexity and specificity of endophytic bacterial networks underscore the importance of microbial diversity in plant health, adaptation, and productivity, opening avenues for agricultural innovation through the manipulation or enhancement of these microbial communities.

**Figure 7:**
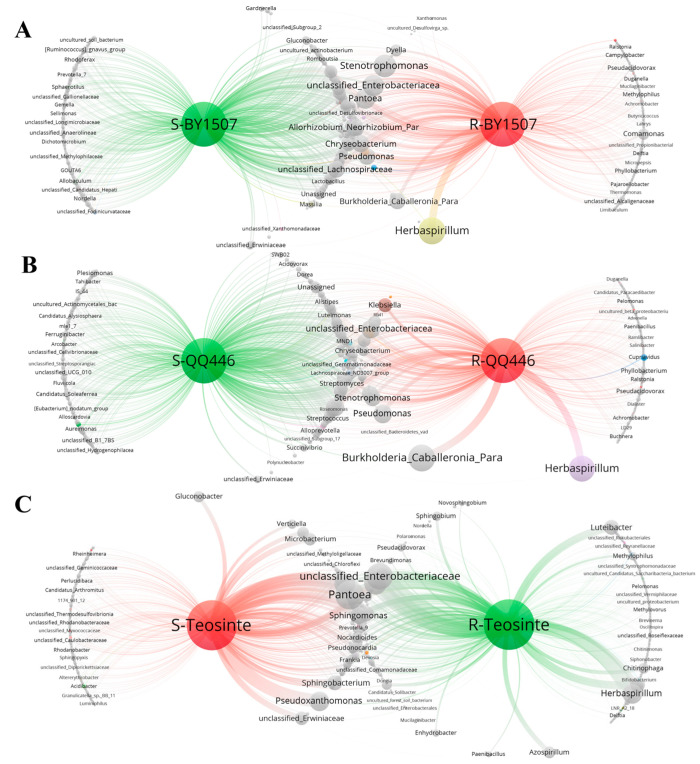
Co-occurrence network analysis of endophytic bacteria with seeds and germinating roots among seed maize (BY1507) (**A**), silage maize (QQ446) (**B**), and wild maize (Teosinte) (**C**).

The co-occurrence network analysis of endophytic bacteria in both the seeds and germinating roots of BY1507, QQ446, and Teosinte (Figure 7) reveals distinct modules represented by different colors, with the entire network partitioned into three main modules.

Figure 7A illustrates the network structure of the endophytic bacteria in BY1507 seeds and germinating roots, comprising 706 genera or nodes interconnected by 1966 edges. Notably, there are 279 common endophytic bacteria shared between BY1507 seeds and their germinating roots, constituting 39.52% of the total endophytes. A total link strength (TLS) analysis highlights *Herbaspirillum* as the most strongly associated genus in endophytic bacteria, followed by *Stenotrophomonas*, *unclassified_Enterobacteriaceae*, *Pantoea*, and *Allorhizobium_Neorhizobium_Pararhizobium_Rhizobium*. These findings underscore the significance of these genera in the endophytic bacterial communities of both BY1507 seeds and germinating roots. Furthermore, unique endophytic bacteria are present in both BY1507 seeds (e.g., *Allobaculum*, *Rhodoferax*, and *Nordella*) and germinating roots (e.g., *Comamonas*, *Pseudacidovorax*, and *unclassified_Alcaligenaceae*), emphasizing the environmental acquisition capability of germinating-root endophytes.

In Figure 7B, the co-occurrence network of QQ446 seed and germinating root endophytic bacteria is depicted, consisting of 717 genera or nodes connected by 1994 edges. This network reveals 282 common endophytic bacteria shared between QQ446 seeds and their germinating roots, accounting for 39.33% of the total endophytes. *Burkholderia_Caballeronia_Paraburkholderia* exhibits the highest association among seed endophytic bacteria, followed by *Herbaspirillum*, *Pseudomonas*, *unclassified_Enterobacteriaceae,* and *unclassified_Lachnospiraceae*. Additionally, numerous unique endophytic bacteria are identified in QQ446 seeds and germinating roots, further highlighting the environmental acquisition capability of germinating root endophytes.

Figure 7C represents the co-occurrence network of Teosinte seed and germinating-root endophytic bacteria, featuring 621 genera or nodes connected by 1718 edges. Within this network, 240 common endophytic bacteria are shared between Teosinte seeds and their germinating roots, accounting for 38.65% of the total endophytes. *Pantoea* is identified as the genus with the highest association in seed endophytic bacteria, followed by *unclassified_Enterobacteriaceae*, *Pseudomonas*, *Allorhizobium_Neorhizobium_Pararhizobium_Rhizobium,* and *Herbaspirillum* according to the TLS analysis. Additionally, Teosinte seeds and their germinating roots exhibit a rich diversity of unique endophytic bacteria.

## 4. Discussion

Seeds are critical reproductive organs in plants and house a complex and abundant microbiome comprising up to 9000 microbial species and as many as two billion bacterial cells [34,40,41]. Existing research has demonstrated that seeds harbor a diverse array of endophytic bacteria [41]. This diversity is essential for the stability and functionality of microbial communities, with the alpha diversity index serving as a measure of their structure, health, and metabolic potential [42,43]. The diversity and abundance of endophytes may be influenced by factors such as genotype and plant developmental stages. Previous research has shown that the occurrence and distribution of endophytic bacteria vary significantly based on maize genotype, plant part, and developmental stage [44,45,46]. However, limited information exists on the effects of different maize varieties on the endophytic bacterial communities in seeds and germinating roots. In this study, the diversity indices of seed endophytic bacteria among BY1507, QQ446, and Teosinte showed no significant differences (*p* > 0.05), suggesting uniform microbial diversity across these maize varieties. Although the Chao1 and ACE indices did not differ significantly between the varieties and root samples, the Shannon and Simpson indices were markedly higher in Teosinte, indicating a greater potential for adaptation or recovery, likely linked to its wild genetic origins. Nonetheless, shifts in the diversity and abundance of bacterial populations in seeds and germinating roots were observed among seed maize (BY1507), silage maize (QQ446), and wild maize (Teosinte).

Plant seeds harbor diverse endophytic bacterial communities, which play a crucial role in forming seedling microbial communities, as observed in rice, tobacco, and maize [47,48,49,50]. Xie et al. utilized Illumina high-throughput sequencing to analyze endophytic bacterial structures in seeds from three tobacco varieties, finding Proteobacteria, Actinobacteria, Firmicutes, and Bacteroidetes as the dominant phyla [48]. Similarly, Hameed noted variations in endophytic bacteria among four rice varieties [51]. In this study, 80% of the endophytic bacteria in seed maize and silage maize seeds, as well as initiation roots, belonged to Proteobacteria, Firmicutes, and Actinobacteriota, mirroring the bacterial composition in their wild ancestor, maize [16]. This suggests a conservation of species composition at the phylum level among maize species. However, at the genus level, significant variations were observed. In the seed samples, the dominant genera included Pantoea, Lachnospiraceae, and Pararhizobium/Rhizobium for BY1507; Lachnospiraceae, Nocardioides, and unclassified_Bacteria for QQ446; and Pantoea, Pararhizobium, and Pseudoxanthomonas for Teosinte. The root samples also showed varied dominant genera, indicating significant genus-level differences in the endophytic bacterial communities of germinating roots across the maize varieties. These findings highlight the influence of genetic factors on the structure of the microbial communities associated with seeds [16,19,21].

Extensive research has shown that seed endophytic bacteria not only enhance seed germination and stimulate plant growth but also increase resistance to various pathogens and environmental stresses [50,51,52,53,54]. These bacteria promote the synthesis and accumulation of bioactive compounds, exerting diverse biological effects [55,56,57,58,59,60]. Notably, *Pantoea* connects human pathogens and plants, offering functions like plant growth promotion, photosynthate allocation, nitrogen fixation, and phosphate solubilization [50,52,53]. This genus has been detected in the seeds of rice, shoots of sugarcane, and roots of cassava [50,52,53]. Among the bacteria involved in phosphate solubilization, *Nocardioides*, *Sphingomonas*, and *Nocardioides* stand out for their ability to solubilize phytate, as evidenced in soybean seeds [51,54,55]. Furthermore, *Pseudomonas* and *Stenotrophomonas* enhance plant growth and alleviate both biotic and abiotic stresses [54,55,56,57]. Recent studies also highlight that *Escherichia-shigella* and *Pseudonocardia* can influence Golgi function and manifest anti-inflammatory effects [58,59,60]. In this study, there were 416 bacterial genera identified in the seeds of BY1507, QQ446, and Teosinte, which suggests a substantial potential for environmental adaptability. Previous studies have indicated that *Pantoea*, as an endophyte, supports functions such as nitrogen fixation, phosphorus enhancement, and the promotion of plant growth and photosynthetic activity in rice and reed seeds [50,52,53]. Nocardioides and Sphingomonas produce phytate-solubilizing enzymes with antioxidant and anti-inflammatory properties, boosting plant resilience to stress [51]. Additionally, *Pseudomonas* and *Stenotrophomonas* are known to bolster plant growth and mitigate both biotic and abiotic stresses [54,55,56,57]. Furthermore, this study reveals that different maize varieties host distinct groups of specific endophytic bacteria, such as *Campylobacter*, *Glycomyces*, and *Herbaspirillum,* in their seeds and roots. This diversity is likely due to interactions between root endophytic bacteria and the broader microbial community in the rhizosphere and environment, resulting in a more complex root endophytic bacterial community and functionality.

Seed endophytes, crucial to plant microbial ecosystems, serve both as the final stage for microbial transmission in seeds and the initial phase for microbial community development in germinating seedlings [61]. These endophytes often outperform soil-borne microbes in colonizing root surfaces during seedling growth, thus playing a significant role in the rhizosphere. Despite their critical importance, detailed studies on seed endophytes are limited, mainly due to the absence of in-depth molecular insights into seed–endophyte interactions and the scant genomic data on uncultivable strains. Future research should focus on unraveling the molecular mechanisms of seed–endophyte interactions using advanced techniques, such as proteomics and metabolomics. Additionally, metagenomic approaches could help explore the diversity and functional attributes of endophytes, particularly for identifying less diverse communities like those in seeds.

## 5. Conclusions

The comprehensive analysis revealed the presence of 18 bacterial phyla, with Proteobacteria, Firmicutes, Actinobacteriota, and Bacteroidota being predominant across both seeds and germinating roots. At the genus level, 416 bacterial genera were identified, among which *Pantoea*, *Lachnospiraceae*, *Pararhizobium/Rhizobium*, *Enterobacteriaceae*, *Stenotrophomonas*, and *Pseudonocardia* were notably prevalent, each exhibiting a relative abundance exceeding 10%. Furthermore, the diversity indices of seed endophytes among BY1507, QQ446, and Teosinte revealed no significant differences. However, the Shannon and Simpson indices for the endophytes of germinating roots in the wild-variety Teosinte were significantly higher compared to those in the domesticated varieties of BY1507 and QQ446. PCoA demonstrated a significant overlap in the endophytic bacterial communities from the seeds of BY1507, QQ446, and Teosinte, though distinct clustering patterns emerged. A co-occurrence network analysis indicated that more than 30% of endophytic bacteria are shared among BY1507, QQ446, and Teosinte across seeds and germinating roots. In conclusion, this study analyzed the difference in endophytic bacterial communities within the seeds and germinating roots of seed maize, silage maize, and wild maize, providing foundational insights for future studies into the formation, physiological responses, and ecological adaptations of these endophytic bacterial communities.

## Data Availability

The original contributions presented in the study are included in the article, further inquiries can be directed to the corresponding authors.
